# [*N*,*N*-Bis(diphenyl­phosphino)propyl­amine-κ^2^ *P*,*P*]bromidotricarbonyl­rhenium(I)

**DOI:** 10.1107/S1600536809047242

**Published:** 2009-11-14

**Authors:** Marietjie Schutte, Hendrik G. Visser, Alice Brink

**Affiliations:** a Department of Chemistry, University of the Free State, PO Box 339, Bloemfontein 9330, South Africa

## Abstract

In the title compound, [ReBr(C_27_H_27_NP_2_)(CO)_3_], the Re^I^ atom is octa­hedrally surrounded by three carbonyl ligands in a facial arrangement, a bromide ligand and the *P*,*P*′-bidentate ligand Bis(diphenyl­phosphino)propyl­amine. The compound exhibits substitutional disorder of the bromide ligand and the axial carbonyl ligand, with almost 50% occupancy for both Br amd CO [0.538 (4) and 0.462 (4), respectively]. In addition, the propyl chain on the N atom of the bidentate ligand exhibits a 0.648 (9):0.352 (9) disorder. C—H⋯O and C—H⋯Br hydrogen bonding consolidates the crystal packing.

## Related literature

For the synthesis of the Re^I^-tricarbonyl synthon: Alberto *et al.* (1996 ▶). For the synthesis and structures of related complexes: Graziani & Casellato (1996 ▶); Kemp (2006 ▶); Mundwiler *et al.* (2004 ▶); Rossi *et al.* (1993 ▶); Schutte & Visser (2008 ▶); Schutte & *et al.* (2007 ▶, 2008 ▶); Steil *et al.* (1989 ▶).
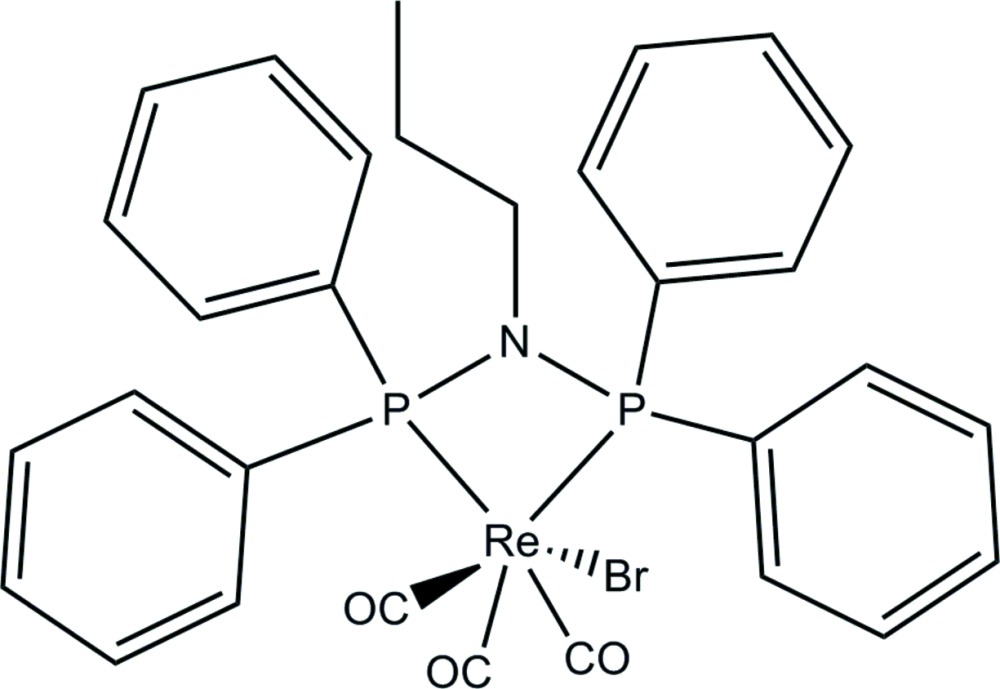


## Experimental

### 

#### Crystal data

[ReBr(C_27_H_27_NP_2_)(CO)_3_]
*M* *_r_* = 777.58
Monoclinic, 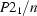

*a* = 11.0120 (2) Å
*b* = 17.1620 (3) Å
*c* = 15.2090 (2) Å
β = 96.735 (2)°
*V* = 2854.48 (7) Å^3^
*Z* = 4
Mo *K*α radiation
μ = 5.80 mm^−1^
*T* = 100 K
0.10 × 0.08 × 0.05 mm

#### Data collection

Oxford Diffraction Xcalibur 3 CCD area-detector diffractometer
Absorption correction: multi-scan (*CrysAlis RED*; Oxford Diffraction, 2006 ▶) *T*_min_ = 0.595, *T*_max_ = 0.760
22483 measured reflections
6863 independent reflections
5197 reflections with *I* > 2σ(*I*)
*R*_int_ = 0.046

#### Refinement

*R*[*F* ^2^ > 2σ(*F* ^2^)] = 0.033
*wR*(*F* ^2^) = 0.088
*S* = 1.03
6863 reflections
387 parameters
H-atom parameters constrained
Δρ_max_ = 1.95 e Å^−3^
Δρ_min_ = −1.11 e Å^−3^

### 

Data collection: *CrysAlis CCD* (Oxford Diffraction, 2006 ▶); cell refinement: *CrysAlis RED* (Oxford Diffraction, 2006 ▶); data reduction: *CrysAlis RED*; program(s) used to solve structure: *SHELXS97* (Sheldrick, 2008 ▶); program(s) used to refine structure: *SHELXL97* (Sheldrick, 2008 ▶); molecular graphics: *DIAMOND* (Brandenberg & Putz, 2004 ▶); software used to prepare material for publication: *WinGX* (Farrugia, 1999 ▶).

## Supplementary Material

Crystal structure: contains datablocks global, I. DOI: 10.1107/S1600536809047242/fi2092sup1.cif

Structure factors: contains datablocks I. DOI: 10.1107/S1600536809047242/fi2092Isup2.hkl

Additional supplementary materials:  crystallographic information; 3D view; checkCIF report

## Figures and Tables

**Table:** 

Re1—C3*B*	1.866 (15)
Re1—C2	1.952 (5)
Re1—C1	1.962 (6)
Re1—C3*A*	1.968 (18)
Re1—P2	2.4375 (14)
Re1—P1	2.4583 (15)
Re1—Br2	2.617 (3)
Re1—Br1	2.619 (2)

**Table:** 

P2—Re1—P1	66.65 (4)

**Table 2:** Hydrogen-bond geometry (Å, °)

*D*—H⋯*A*	*D*—H	H⋯*A*	*D*⋯*A*	*D*—H⋯*A*
C12—H12⋯O3*B*	0.95	2.51	3.39 (2)	155
C14—H14⋯Br1^i^	0.95	2.81	3.524 (6)	133
C46—H46⋯Br1	0.95	2.63	3.519 (6)	157

Symmetry code: (i) 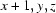.

**Fig. 1.**
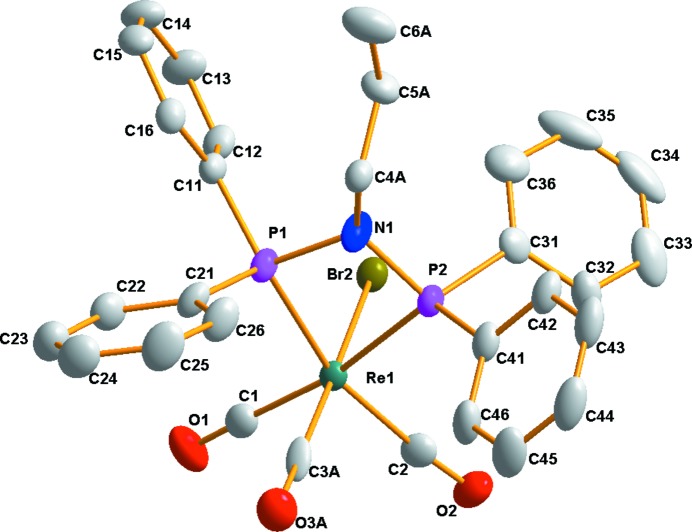
Representation of the title compound, showing the numbering scheme and displacement ellipsoids (50% probability). One of the disordered propyl groups are omitted for clarity. Hydrogen atoms omitted for the same reason.

## supplementary crystallographic information

### Comment

The Re—CO bond distances of 1.866 (15) Å to 1.968 (18) Å are well within the normal range (Mundwiler *et al.*, 2004, Kemp (2006), Schutte *et al.* (2008).) The rhenium(I) bromido bond distance of 2.617 (2) Å compare well with related structures (Schutte *et al.* (2007), Graziani *et al.* (1996)). The P1—Re—P2 bite angle of 66.65 (4) ° is almost identical to similar structures (Rossi *et al.* (1993), Steil *et al.* (1989) and Graziani *et al.* (1996)). The octahedral arrangement around the rhenium atom is slightly distorted, possibly due to the small bite angle of the bidentate ligand. Three types of intramolecular and intermolecular hydrogen bonds are observed and listed in Table 2. The compound exhibits substitutional disorder of the bromido ligand and the axial carbonyl ligand, with 53.8% occupancy for both Br amd CO. Also, the propyl chain on the nitrogen atom of the bidentate ligand exhibits a 64.8/35.2% disorder.

### Experimental

[NEt_4_]_2_[Re(CO)_3_Br_3_] (100 mg, 0.130 mmol), as prepared by Alberto *et al.* (1996), was dissolved in 10 ml of methanol. From here, the reaction was done under a nitrogen atmosphere. The reaction mixture was heated to 37°C. Bis(diphenylphosphino)-propylamine (66.58 mg, 0.156 mmol) was added to the reaction mixture, heated to 50°C and stirred for 1 h. It was left to cool down and the precipitate was filtered off and dried under vacuum. A 84.71% yield (85.5 mg, 0.1099 mmol) was obtained. Crystals, suitable for X-ray diffraction data collection were obtained by recrystalizing from methanol.

### Refinement

The aromatic H atoms were placed in geometrically idealized positions and constrained to ride on its parent atoms with *U*_iso_(H) = 1.2*U*_eq_(C). The aliphatic H atoms were place in geometrically idealized positions and constrained to ride on its parent atoms with *U*_iso_(H) = 1.2*U*_eq_(C) for methylene carbon atoms and *U*_iso_(H) = 1.5*U*_eq_(C) for methyl atoms. The same procedures were used for the H atom treatment of the disordered part of the molecule by using. The PART instruction was used to create both the disordered parts of the propyl group and the occupancies were determined with a interconnected free variable instruction. The highest and lowest electron density peak and hole lies within 0.89 and 0.79 Å from Re1 respectively.

### Crystal data

**Table:**

[ReBr(C_27_H_27_NP_2_)(CO)_3_]	*F*(000) = 1512
*M**_r_* = 777.58	*D*_x_ = 1.809 Mg m^−^^3^
Monoclinic, *P*2_1_/*n*	Mo *K*α radiation, λ = 0.71069 Å
Hall symbol: -P 2yn	Cell parameters from 10593 reflections
*a* = 11.0120 (2) Å	θ = 2.2–33.2°
*b* = 17.1620 (3) Å	µ = 5.80 mm^−^^1^
*c* = 15.2090 (2) Å	*T* = 100 K
β = 96.735 (2)°	Cuboid, colourless
*V* = 2854.48 (7) Å^3^	0.1 × 0.08 × 0.05 mm
*Z* = 4	

### Data collection

**Table:**

Oxford Diffraction Xcalibur 3 CCD area-detector diffractometer	6863 independent reflections
Radiation source: Enhance (Mo) X-ray Source	5197 reflections with *I* > 2σ(*I*)
graphite	*R*_int_ = 0.046
Detector resolution: 16.1829 pixels mm^-1^	θ_max_ = 28°, θ_min_ = 2.2°
ω scans	*h* = −11→14
Absorption correction: multi-scan (*CrysAlis RED*; Oxford Diffraction, 2006)	*k* = −22→21
*T*_min_ = 0.595, *T*_max_ = 0.760	*l* = −20→16
22483 measured reflections	

### Refinement

**Table:**

Refinement on *F*^2^	Primary atom site location: structure-invariant direct methods
Least-squares matrix: full	Secondary atom site location: difference Fourier map
*R*[*F*^2^ > 2σ(*F*^2^)] = 0.033	H-atom parameters constrained
*wR*(*F*^2^) = 0.088	*w* = 1/[σ^2^(*F*_o_^2^) + (0.0476*P*)^2^] where *P* = (*F*_o_^2^ + 2*F*_c_^2^)/3
*S* = 1.02	(Δ/σ)_max_ = 0.001
6863 reflections	Δρ_max_ = 1.95 e Å^−^^3^
387 parameters	Δρ_min_ = −1.11 e Å^−^^3^
0 restraints	

### Special details

**Table:**

Experimental. The intensity data was collected on a Oxford Diffraction Xcalibur 3 area detector diffractometer using an exposure time of 30 s/frame (Oxford, 2006*a*). A total of 552 frames were collected with a frame width of 0.75° covering up to θ = 28.0° with 99.5% completeness accomplized.CrysAlis RED (Oxford Diffraction Ltd, Version 1.171.31.5 (Oxford, 2006b) Empirical absorption correction using spherical harmonics, implemented in SCALE3 ABSPACK scaling algorithm.
Geometry. All e.s.d.'s (except the e.s.d. in the dihedral angle between two l.s. planes) are estimated using the full covariance matrix. The cell e.s.d.'s are taken into account individually in the estimation of e.s.d.'s in distances, angles and torsion angles; correlations between e.s.d.'s in cell parameters are only used when they are defined by crystal symmetry. An approximate (isotropic) treatment of cell e.s.d.'s is used for estimating e.s.d.'s involving l.s. planes.

### Fractional atomic coordinates and isotropic or equivalent isotropic displacement parameters (Å^2^)

**Table:**

	*x*	*y*	*z*	*U*_iso_*/*U*_eq_	Occ. (<1)
Re1	0.686711 (18)	0.106550 (11)	0.202197 (12)	0.02066 (7)	
P2	0.73189 (12)	0.20128 (8)	0.32094 (8)	0.0239 (3)	
P1	0.85557 (12)	0.19498 (7)	0.17874 (8)	0.0242 (3)	
C2	0.5544 (5)	0.0550 (3)	0.2545 (3)	0.0285 (12)	
O2	0.4813 (4)	0.0257 (2)	0.2910 (3)	0.0411 (10)	
C34	0.8309 (8)	0.0726 (4)	0.5839 (4)	0.054 (2)	
H34	0.849	0.0436	0.6371	0.064*	
C35	0.9232 (8)	0.0939 (3)	0.5363 (5)	0.059 (2)	
H35	1.0051	0.0798	0.5564	0.071*	
O1	0.6682 (4)	−0.0050 (2)	0.0405 (2)	0.0438 (10)	
C1	0.6767 (5)	0.0357 (3)	0.1004 (3)	0.0295 (12)	
C32	0.6847 (6)	0.1351 (3)	0.4772 (3)	0.0330 (13)	
H32	0.6024	0.1488	0.4576	0.04*	
C44	0.4952 (6)	0.4065 (3)	0.3942 (4)	0.0443 (17)	
H44	0.4454	0.4483	0.4098	0.053*	
C46	0.5319 (6)	0.3030 (3)	0.2950 (4)	0.0370 (14)	
H46	0.5082	0.2743	0.2424	0.044*	
C31	0.7784 (5)	0.1570 (3)	0.4284 (3)	0.0271 (11)	
C11	1.0162 (5)	0.1714 (3)	0.1741 (3)	0.0243 (11)	
C26	0.7771 (5)	0.3402 (3)	0.1033 (4)	0.0368 (14)	
H26	0.7563	0.3547	0.1599	0.044*	
C22	0.8530 (5)	0.2457 (3)	0.0066 (4)	0.0332 (13)	
H22	0.8869	0.196	−0.003	0.04*	
C21	0.8280 (5)	0.2662 (3)	0.0915 (3)	0.0273 (12)	
C13	1.1832 (5)	0.0804 (4)	0.1978 (4)	0.0382 (14)	
H13	1.2126	0.0295	0.2129	0.046*	
C16	1.0980 (5)	0.2284 (3)	0.1530 (3)	0.0257 (11)	
H16	1.0689	0.2792	0.1373	0.031*	
C41	0.6376 (5)	0.2829 (3)	0.3499 (3)	0.0281 (12)	
C36	0.8976 (6)	0.1362 (3)	0.4586 (4)	0.0413 (15)	
H36	0.9623	0.151	0.4258	0.05*	
C15	1.2208 (5)	0.2120 (3)	0.1548 (3)	0.0313 (12)	
H15	1.2759	0.2514	0.1407	0.038*	
C14	1.2640 (5)	0.1374 (4)	0.1772 (4)	0.0396 (14)	
H14	1.3485	0.1258	0.1784	0.048*	
N1	0.8534 (5)	0.2416 (3)	0.2781 (3)	0.0355 (12)	
C45	0.4617 (6)	0.3653 (4)	0.3180 (4)	0.0447 (16)	
H45	0.3899	0.3795	0.2807	0.054*	
C24	0.7837 (6)	0.3709 (4)	−0.0495 (4)	0.0439 (15)	
H24	0.771	0.407	−0.097	0.053*	
C12	1.0608 (5)	0.0966 (3)	0.1967 (3)	0.0287 (12)	
H12	1.0062	0.0571	0.2113	0.034*	
C25	0.7571 (6)	0.3919 (3)	0.0334 (4)	0.0454 (16)	
H25	0.7248	0.4421	0.0427	0.054*	
C23	0.8290 (5)	0.2972 (4)	−0.0641 (4)	0.0407 (14)	
H23	0.8436	0.282	−0.122	0.049*	
C42	0.6723 (5)	0.3252 (3)	0.4263 (4)	0.0322 (13)	
H42	0.7448	0.3115	0.4632	0.039*	
C43	0.6020 (6)	0.3875 (3)	0.4492 (4)	0.0408 (15)	
H43	0.626	0.4168	0.5013	0.049*	
C33	0.7130 (8)	0.0925 (3)	0.5559 (4)	0.0489 (19)	
H33	0.6496	0.0776	0.5897	0.059*	
Br2	0.8260 (2)	0.00301 (16)	0.29098 (18)	0.0268 (5)	0.462 (4)
C3A	0.5759 (17)	0.1762 (8)	0.1278 (10)	0.027 (2)	0.462 (4)
O3A	0.5100 (18)	0.2120 (12)	0.0826 (14)	0.041 (6)	0.462 (4)
Br1	0.5387 (2)	0.19542 (14)	0.09865 (15)	0.0254 (5)	0.538 (4)
C3B	0.7961 (12)	0.0397 (7)	0.2678 (8)	0.027 (2)	0.538 (4)
O3B	0.8632 (15)	−0.0078 (12)	0.3052 (13)	0.055 (6)	0.538 (4)
C4B	0.9665 (16)	0.2791 (10)	0.3419 (10)	0.026 (4)	0.352 (9)
H4B1	0.9494	0.278	0.4043	0.031*	0.352 (9)
H4B2	1.0424	0.2494	0.337	0.031*	0.352 (9)
C5B	0.9802 (16)	0.3627 (10)	0.3113 (11)	0.0342 (18)	0.352 (9)
H5B1	0.9909	0.3637	0.2476	0.041*	0.352 (9)
H5B2	0.9061	0.3931	0.3198	0.041*	0.352 (9)
C6B	1.090 (4)	0.3979 (17)	0.365 (3)	0.035 (7)	0.352 (9)
H6B1	1.1007	0.4517	0.3454	0.052*	0.352 (9)
H6B2	1.1631	0.3675	0.356	0.052*	0.352 (9)
H6B3	1.0783	0.3975	0.4276	0.052*	0.352 (9)
C4A	0.9176 (8)	0.3160 (5)	0.2997 (5)	0.025 (2)	0.648 (9)
H4A1	0.8637	0.3516	0.3285	0.03*	0.648 (9)
H4A2	0.9376	0.341	0.2445	0.03*	0.648 (9)
C5A	1.0330 (10)	0.3019 (5)	0.3604 (6)	0.0342 (18)	0.648 (9)
H5A1	1.0798	0.2593	0.3362	0.041*	0.648 (9)
H5A2	1.012	0.2852	0.419	0.041*	0.648 (9)
C6A	1.112 (2)	0.3747 (10)	0.3713 (18)	0.046 (4)	0.648 (9)
H6A1	1.1864	0.3636	0.4113	0.069*	0.648 (9)
H6A2	1.0663	0.4167	0.3961	0.069*	0.648 (9)
H6A3	1.1344	0.3907	0.3134	0.069*	0.648 (9)

### Atomic displacement parameters (Å^2^)

**Table:**

	*U*^11^	*U*^22^	*U*^33^	*U*^12^	*U*^13^	*U*^23^
Re1	0.02282 (11)	0.01788 (10)	0.02159 (10)	0.00267 (9)	0.00387 (7)	0.00426 (8)
P2	0.0250 (7)	0.0247 (7)	0.0234 (6)	0.0020 (6)	0.0084 (5)	0.0003 (5)
P1	0.0275 (7)	0.0192 (7)	0.0278 (7)	−0.0005 (5)	0.0113 (6)	−0.0025 (5)
C2	0.032 (3)	0.021 (3)	0.033 (3)	0.004 (2)	0.001 (2)	0.009 (2)
O2	0.032 (2)	0.045 (3)	0.047 (2)	−0.004 (2)	0.0088 (19)	0.017 (2)
C34	0.100 (6)	0.021 (3)	0.032 (3)	0.007 (4)	−0.025 (4)	−0.006 (3)
C35	0.072 (5)	0.025 (4)	0.068 (5)	0.000 (3)	−0.043 (4)	−0.002 (3)
O1	0.064 (3)	0.035 (2)	0.030 (2)	0.011 (2)	−0.004 (2)	−0.0039 (19)
C1	0.031 (3)	0.029 (3)	0.029 (3)	0.004 (2)	0.004 (2)	0.009 (2)
C32	0.051 (4)	0.022 (3)	0.026 (3)	0.009 (3)	0.010 (3)	0.004 (2)
C44	0.063 (4)	0.028 (3)	0.047 (4)	0.016 (3)	0.031 (3)	0.013 (3)
C46	0.046 (4)	0.039 (3)	0.029 (3)	0.014 (3)	0.016 (3)	0.009 (2)
C31	0.037 (3)	0.019 (3)	0.026 (3)	0.005 (2)	0.004 (2)	−0.007 (2)
C11	0.025 (3)	0.026 (3)	0.023 (2)	0.000 (2)	0.006 (2)	−0.004 (2)
C26	0.044 (4)	0.021 (3)	0.049 (4)	0.007 (3)	0.020 (3)	0.002 (2)
C22	0.035 (3)	0.027 (3)	0.038 (3)	0.007 (2)	0.008 (3)	−0.002 (2)
C21	0.026 (3)	0.020 (3)	0.039 (3)	0.002 (2)	0.014 (2)	0.003 (2)
C13	0.030 (3)	0.031 (3)	0.053 (4)	0.006 (3)	0.002 (3)	0.011 (3)
C16	0.033 (3)	0.018 (2)	0.026 (3)	−0.001 (2)	0.007 (2)	−0.001 (2)
C41	0.038 (3)	0.021 (3)	0.028 (3)	0.002 (2)	0.015 (2)	0.004 (2)
C36	0.046 (4)	0.028 (3)	0.048 (4)	−0.002 (3)	−0.004 (3)	−0.007 (3)
C15	0.028 (3)	0.032 (3)	0.033 (3)	−0.010 (2)	0.001 (2)	0.004 (2)
C14	0.025 (3)	0.043 (3)	0.049 (4)	−0.002 (3)	−0.003 (3)	0.014 (3)
N1	0.041 (3)	0.030 (3)	0.039 (3)	−0.012 (2)	0.020 (2)	−0.017 (2)
C45	0.058 (4)	0.039 (3)	0.039 (3)	0.025 (3)	0.017 (3)	0.017 (3)
C24	0.045 (4)	0.036 (3)	0.050 (4)	0.006 (3)	0.008 (3)	0.016 (3)
C12	0.032 (3)	0.023 (3)	0.033 (3)	−0.005 (2)	0.009 (2)	0.000 (2)
C25	0.056 (4)	0.024 (3)	0.059 (4)	0.013 (3)	0.018 (3)	0.014 (3)
C23	0.038 (3)	0.047 (4)	0.039 (3)	0.007 (3)	0.009 (3)	0.008 (3)
C42	0.042 (3)	0.022 (3)	0.036 (3)	0.002 (2)	0.020 (3)	0.002 (2)
C43	0.064 (4)	0.026 (3)	0.038 (3)	0.002 (3)	0.029 (3)	0.006 (3)
C33	0.098 (6)	0.018 (3)	0.033 (3)	0.006 (3)	0.016 (4)	−0.004 (2)
Br2	0.0311 (11)	0.0207 (10)	0.0296 (9)	0.0008 (9)	0.0076 (8)	0.0047 (7)
C3A	0.049 (6)	0.009 (4)	0.028 (5)	−0.007 (4)	0.021 (4)	0.000 (4)
O3A	0.047 (12)	0.032 (10)	0.044 (10)	0.011 (7)	0.006 (7)	0.007 (6)
Br1	0.0226 (10)	0.0286 (12)	0.0253 (9)	0.0057 (7)	0.0049 (7)	0.0087 (7)
C3B	0.049 (6)	0.009 (4)	0.028 (5)	−0.007 (4)	0.021 (4)	0.000 (4)
O3B	0.064 (13)	0.042 (8)	0.063 (10)	0.006 (8)	0.024 (8)	0.006 (6)
C4B	0.029 (10)	0.025 (9)	0.023 (8)	0.003 (8)	−0.001 (7)	−0.001 (7)
C5B	0.038 (5)	0.029 (4)	0.034 (4)	−0.006 (4)	−0.004 (4)	−0.004 (3)
C6B	0.047 (18)	0.014 (15)	0.040 (12)	−0.004 (11)	−0.014 (11)	0.009 (11)
C4A	0.027 (5)	0.022 (4)	0.026 (4)	0.000 (4)	0.008 (4)	−0.001 (4)
C5A	0.038 (5)	0.029 (4)	0.034 (4)	−0.006 (4)	−0.004 (4)	−0.004 (3)
C6A	0.050 (9)	0.024 (10)	0.059 (9)	0.006 (8)	−0.020 (6)	0.004 (8)

### Geometric parameters (Å, °)

**Table:**

Re1—C3B	1.866 (15)	C13—H13	0.95
Re1—C2	1.952 (5)	C16—C15	1.379 (7)
Re1—C1	1.962 (6)	C16—H16	0.95
Re1—C3A	1.968 (18)	C41—C42	1.385 (8)
Re1—P2	2.4375 (14)	C36—H36	0.95
Re1—P1	2.4583 (15)	C15—C14	1.394 (8)
Re1—Br2	2.617 (3)	C15—H15	0.95
Re1—Br1	2.619 (2)	C14—H14	0.95
P2—N1	1.702 (5)	N1—C4A	1.478 (9)
P2—C31	1.820 (5)	N1—C4B	1.619 (18)
P2—C41	1.828 (5)	C45—H45	0.95
P2—P1	2.6897 (18)	C24—C25	1.376 (9)
P1—N1	1.713 (4)	C24—C23	1.386 (8)
P1—C21	1.804 (5)	C24—H24	0.95
P1—C11	1.824 (5)	C12—H12	0.95
C2—O2	1.146 (6)	C25—H25	0.95
C34—C33	1.361 (10)	C23—H23	0.95
C34—C35	1.366 (10)	C42—C43	1.388 (8)
C34—H34	0.95	C42—H42	0.95
C35—C36	1.387 (9)	C43—H43	0.95
C35—H35	0.95	C33—H33	0.95
O1—C1	1.142 (6)	C3A—O3A	1.12 (2)
C32—C31	1.392 (7)	C3B—O3B	1.20 (2)
C32—C33	1.406 (8)	C4B—C5B	1.52 (2)
C32—H32	0.95	C4B—H4B1	0.99
C44—C45	1.370 (9)	C4B—H4B2	0.99
C44—C43	1.400 (9)	C5B—C6B	1.50 (4)
C44—H44	0.95	C5B—H5B1	0.99
C46—C45	1.388 (8)	C5B—H5B2	0.99
C46—C41	1.393 (8)	C6B—H6B1	0.98
C46—H46	0.95	C6B—H6B2	0.98
C31—C36	1.386 (8)	C6B—H6B3	0.98
C11—C16	1.392 (7)	C4A—C5A	1.500 (13)
C11—C12	1.402 (7)	C4A—H4A1	0.99
C26—C25	1.382 (8)	C4A—H4A2	0.99
C26—C21	1.408 (7)	C5A—C6A	1.52 (3)
C26—H26	0.95	C5A—H5A1	0.99
C22—C23	1.394 (8)	C5A—H5A2	0.99
C22—C21	1.395 (7)	C6A—H6A1	0.98
C22—H22	0.95	C6A—H6A2	0.98
C13—C12	1.375 (7)	C6A—H6A3	0.98
C13—C14	1.383 (8)		
			
C3B—Re1—C2	88.2 (3)	C15—C16—H16	119.6
C3B—Re1—C1	90.8 (3)	C11—C16—H16	119.6
C2—Re1—C1	93.7 (2)	C42—C41—C46	120.2 (5)
C3B—Re1—C3A	177.1 (5)	C42—C41—P2	119.6 (4)
C2—Re1—C3A	93.9 (5)	C46—C41—P2	120.2 (4)
C1—Re1—C3A	87.1 (4)	C31—C36—C35	120.4 (7)
C3B—Re1—P2	87.2 (3)	C31—C36—H36	119.8
C2—Re1—P2	95.62 (16)	C35—C36—H36	119.8
C1—Re1—P2	170.40 (16)	C16—C15—C14	119.9 (5)
C3A—Re1—P2	94.6 (4)	C16—C15—H15	120.1
C3B—Re1—P1	90.3 (3)	C14—C15—H15	120.1
C2—Re1—P1	162.26 (16)	C13—C14—C15	119.7 (5)
C1—Re1—P1	104.00 (16)	C13—C14—H14	120.2
C3A—Re1—P1	88.3 (4)	C15—C14—H14	120.2
P2—Re1—P1	66.65 (4)	C4A—N1—P2	130.1 (4)
C3B—Re1—Br2	5.0 (3)	C4B—N1—P2	121.1 (6)
C2—Re1—Br2	84.41 (16)	C4A—N1—P1	122.9 (4)
C1—Re1—Br2	87.79 (16)	C4B—N1—P1	128.7 (6)
C3A—Re1—Br2	174.5 (4)	P2—N1—P1	103.9 (2)
P2—Re1—Br2	90.82 (7)	C44—C45—C46	120.5 (6)
P1—Re1—Br2	94.96 (7)	C44—C45—H45	119.7
C3B—Re1—Br1	175.4 (3)	C46—C45—H45	119.7
C2—Re1—Br1	93.86 (16)	C25—C24—C23	120.3 (6)
C1—Re1—Br1	85.02 (16)	C25—C24—H24	119.9
C3A—Re1—Br1	2.1 (4)	C23—C24—H24	119.9
P2—Re1—Br1	96.64 (7)	C13—C12—C11	120.2 (5)
P1—Re1—Br1	88.92 (7)	C13—C12—H12	119.9
Br2—Re1—Br1	172.48 (9)	C11—C12—H12	119.9
N1—P2—C31	111.2 (3)	C24—C25—C26	120.3 (5)
N1—P2—C41	106.0 (2)	C24—C25—H25	119.8
C31—P2—C41	102.2 (2)	C26—C25—H25	119.8
N1—P2—Re1	95.10 (15)	C24—C23—C22	119.6 (6)
C31—P2—Re1	113.44 (16)	C24—C23—H23	120.2
C41—P2—Re1	128.11 (19)	C22—C23—H23	120.2
C31—P2—P1	126.09 (18)	C41—C42—C43	120.4 (6)
C41—P2—P1	125.45 (16)	C41—C42—H42	119.8
Re1—P2—P1	57.05 (4)	C43—C42—H42	119.8
N1—P1—C21	108.3 (2)	C42—C43—C44	118.9 (6)
N1—P1—C11	104.6 (2)	C42—C43—H43	120.5
C21—P1—C11	101.6 (2)	C44—C43—H43	120.5
N1—P1—Re1	94.08 (16)	C34—C33—C32	120.4 (7)
C21—P1—Re1	117.41 (18)	C34—C33—H33	119.8
C11—P1—Re1	128.48 (17)	C32—C33—H33	119.8
C21—P1—P2	120.47 (17)	O3A—C3A—Re1	176 (2)
C11—P1—P2	128.68 (17)	O3B—C3B—Re1	174.9 (14)
Re1—P1—P2	56.30 (4)	C5B—C4B—N1	106.8 (12)
O2—C2—Re1	175.1 (5)	C5B—C4B—H4B1	110.4
C33—C34—C35	120.5 (6)	N1—C4B—H4B1	110.4
C33—C34—H34	119.7	C5B—C4B—H4B2	110.4
C35—C34—H34	119.7	N1—C4B—H4B2	110.4
C34—C35—C36	120.2 (7)	H4B1—C4B—H4B2	108.6
C34—C35—H35	119.9	C6B—C5B—C4B	108.5 (16)
C36—C35—H35	119.9	C6B—C5B—H5B1	110
O1—C1—Re1	178.4 (5)	C4B—C5B—H5B1	110
C31—C32—C33	119.3 (6)	C6B—C5B—H5B2	110
C31—C32—H32	120.3	C4B—C5B—H5B2	110
C33—C32—H32	120.3	H5B1—C5B—H5B2	108.4
C45—C44—C43	120.6 (6)	C5B—C6B—H6B1	109.5
C45—C44—H44	119.7	C5B—C6B—H6B2	109.5
C43—C44—H44	119.7	H6B1—C6B—H6B2	109.5
C45—C46—C41	119.3 (6)	C5B—C6B—H6B3	109.5
C45—C46—H46	120.3	H6B1—C6B—H6B3	109.5
C41—C46—H46	120.3	H6B2—C6B—H6B3	109.5
C36—C31—C32	119.1 (5)	N1—C4A—C5A	110.2 (7)
C36—C31—P2	124.1 (4)	N1—C4A—H4A1	109.6
C32—C31—P2	116.3 (4)	C5A—C4A—H4A1	109.6
C16—C11—C12	118.9 (5)	N1—C4A—H4A2	109.6
C16—C11—P1	120.6 (4)	C5A—C4A—H4A2	109.6
C12—C11—P1	120.4 (4)	H4A1—C4A—H4A2	108.1
C25—C26—C21	120.7 (5)	C4A—C5A—C6A	111.5 (9)
C25—C26—H26	119.7	C4A—C5A—H5A1	109.3
C21—C26—H26	119.7	C6A—C5A—H5A1	109.3
C23—C22—C21	120.9 (5)	C4A—C5A—H5A2	109.3
C23—C22—H22	119.5	C6A—C5A—H5A2	109.3
C21—C22—H22	119.5	H5A1—C5A—H5A2	108
C22—C21—C26	118.1 (5)	C5A—C6A—H6A1	109.5
C22—C21—P1	118.4 (4)	C5A—C6A—H6A2	109.5
C26—C21—P1	123.5 (4)	H6A1—C6A—H6A2	109.5
C12—C13—C14	120.6 (6)	C5A—C6A—H6A3	109.5
C12—C13—H13	119.7	H6A1—C6A—H6A3	109.5
C14—C13—H13	119.7	H6A2—C6A—H6A3	109.5
C15—C16—C11	120.7 (5)		
			
C3B—Re1—P2—N1	88.4 (4)	N1—P1—C11—C12	98.5 (4)
C2—Re1—P2—N1	176.3 (2)	C21—P1—C11—C12	−148.8 (4)
C3A—Re1—P2—N1	−89.3 (5)	Re1—P1—C11—C12	−8.9 (5)
P1—Re1—P2—N1	−3.19 (18)	P2—P1—C11—C12	65.3 (5)
Br2—Re1—P2—N1	91.86 (19)	C23—C22—C21—C26	0.4 (8)
Br1—Re1—P2—N1	−89.13 (19)	C23—C22—C21—P1	177.4 (5)
C3B—Re1—P2—C31	−27.4 (4)	C25—C26—C21—C22	−2.4 (9)
C2—Re1—P2—C31	60.6 (2)	C25—C26—C21—P1	−179.3 (5)
C3A—Re1—P2—C31	154.9 (5)	N1—P1—C21—C22	165.6 (4)
P1—Re1—P2—C31	−119.0 (2)	C11—P1—C21—C22	55.7 (5)
Br2—Re1—P2—C31	−23.9 (2)	Re1—P1—C21—C22	−89.6 (4)
Br1—Re1—P2—C31	155.1 (2)	P2—P1—C21—C22	−154.8 (4)
C3B—Re1—P2—C41	−156.7 (4)	N1—P1—C21—C26	−17.6 (6)
C2—Re1—P2—C41	−68.8 (3)	C11—P1—C21—C26	−127.4 (5)
C3A—Re1—P2—C41	25.6 (5)	Re1—P1—C21—C26	87.2 (5)
P1—Re1—P2—C41	111.7 (2)	P2—P1—C21—C26	22.1 (6)
Br2—Re1—P2—C41	−153.2 (2)	C12—C11—C16—C15	−0.3 (7)
Br1—Re1—P2—C41	25.8 (2)	P1—C11—C16—C15	176.0 (4)
Br1—Re1—P2—C41	25.8 (2)	C45—C46—C41—C42	−0.4 (8)
C3B—Re1—P2—P1	91.5 (3)	C45—C46—C41—P2	−179.8 (4)
C2—Re1—P2—P1	179.50 (15)	N1—P2—C41—C42	−77.5 (4)
C3A—Re1—P2—P1	−86.2 (4)	C31—P2—C41—C42	39.0 (5)
Br2—Re1—P2—P1	95.04 (7)	Re1—P2—C41—C42	172.5 (3)
Br1—Re1—P2—P1	−85.94 (7)	P1—P2—C41—C42	−114.4 (4)
Br1—Re1—P2—P1	−85.94 (7)	N1—P2—C41—C46	101.9 (5)
C3B—Re1—P1—N1	−83.7 (4)	C31—P2—C41—C46	−141.6 (4)
C2—Re1—P1—N1	1.5 (5)	Re1—P2—C41—C46	−8.1 (5)
C1—Re1—P1—N1	−174.5 (2)	P1—P2—C41—C46	65.0 (5)
C3A—Re1—P1—N1	98.9 (4)	C32—C31—C36—C35	0.2 (8)
P2—Re1—P1—N1	3.16 (18)	P2—C31—C36—C35	−171.9 (4)
Br2—Re1—P1—N1	−85.59 (19)	C34—C35—C36—C31	−0.2 (9)
Br1—Re1—P1—N1	100.87 (19)	C11—C16—C15—C14	0.4 (8)
Br1—Re1—P1—N1	100.87 (19)	C12—C13—C14—C15	−0.2 (9)
C3B—Re1—P1—C21	163.2 (4)	C16—C15—C14—C13	−0.1 (9)
C2—Re1—P1—C21	−111.5 (5)	C31—P2—N1—C4A	−78.0 (6)
C1—Re1—P1—C21	72.4 (2)	C41—P2—N1—C4A	32.3 (7)
C3A—Re1—P1—C21	−14.2 (4)	Re1—P2—N1—C4A	164.4 (6)
P2—Re1—P1—C21	−109.92 (19)	P1—P2—N1—C4A	160.1 (8)
Br2—Re1—P1—C21	161.3 (2)	C31—P2—N1—C4B	−32.4 (8)
Br1—Re1—P1—C21	−12.22 (19)	C41—P2—N1—C4B	77.9 (8)
Br1—Re1—P1—C21	−12.22 (19)	Re1—P2—N1—C4B	−150.0 (7)
C3B—Re1—P1—C11	28.6 (4)	P1—P2—N1—C4B	−154.3 (9)
C2—Re1—P1—C11	113.8 (5)	C31—P2—N1—P1	121.9 (3)
C1—Re1—P1—C11	−62.3 (3)	C41—P2—N1—P1	−127.7 (3)
C3A—Re1—P1—C11	−148.8 (5)	Re1—P2—N1—P1	4.3 (2)
P2—Re1—P1—C11	115.4 (2)	C21—P1—N1—C4A	−45.5 (6)
Br2—Re1—P1—C11	26.7 (2)	C11—P1—N1—C4A	62.3 (6)
Br1—Re1—P1—C11	−146.9 (2)	Re1—P1—N1—C4A	−166.2 (5)
Br1—Re1—P1—C11	−146.9 (2)	P2—P1—N1—C4A	−161.9 (7)
C3B—Re1—P1—P2	−86.8 (3)	C21—P1—N1—C4B	−92.0 (9)
C2—Re1—P1—P2	−1.6 (5)	C11—P1—N1—C4B	15.8 (9)
C1—Re1—P1—P2	−177.70 (16)	Re1—P1—N1—C4B	147.4 (8)
C3A—Re1—P1—P2	95.7 (4)	P2—P1—N1—C4B	151.6 (9)
Br2—Re1—P1—P2	−88.75 (7)	C21—P1—N1—P2	116.4 (3)
Br1—Re1—P1—P2	97.70 (7)	C11—P1—N1—P2	−135.8 (3)
Br1—Re1—P1—P2	97.70 (7)	Re1—P1—N1—P2	−4.3 (2)
C31—P2—P1—N1	−78.3 (4)	C43—C44—C45—C46	1.3 (9)
C41—P2—P1—N1	69.0 (4)	C41—C46—C45—C44	−0.4 (9)
Re1—P2—P1—N1	−174.9 (3)	C14—C13—C12—C11	0.3 (9)
N1—P2—P1—C21	−80.7 (4)	C16—C11—C12—C13	0.0 (8)
C31—P2—P1—C21	−159.0 (3)	P1—C11—C12—C13	−176.4 (4)
C41—P2—P1—C21	−11.7 (3)	C23—C24—C25—C26	1.1 (10)
Re1—P2—P1—C21	104.5 (2)	C21—C26—C25—C24	1.7 (10)
N1—P2—P1—C11	59.8 (4)	C25—C24—C23—C22	−3.1 (10)
C31—P2—P1—C11	−18.6 (3)	C21—C22—C23—C24	2.3 (9)
C41—P2—P1—C11	128.7 (3)	C46—C41—C42—C43	0.4 (8)
Re1—P2—P1—C11	−115.1 (2)	P2—C41—C42—C43	179.8 (4)
N1—P2—P1—Re1	174.9 (3)	C41—C42—C43—C44	0.4 (8)
C31—P2—P1—Re1	96.5 (2)	C45—C44—C43—C42	−1.3 (8)
C41—P2—P1—Re1	−116.2 (2)	C35—C34—C33—C32	0.3 (9)
C33—C34—C35—C36	−0.1 (9)	C31—C32—C33—C34	−0.2 (8)
C33—C32—C31—C36	0.0 (8)	C2—Re1—Br1—Br1	0.00 (6)
C33—C32—C31—P2	172.7 (4)	C1—Re1—Br1—Br1	0.00 (6)
N1—P2—C31—C36	−19.3 (5)	C3A—Re1—Br1—Br1	0.0 (14)
C41—P2—C31—C36	−132.0 (5)	P2—Re1—Br1—Br1	0.00 (6)
Re1—P2—C31—C36	86.5 (5)	P1—Re1—Br1—Br1	0.00 (6)
P1—P2—C31—C36	21.2 (6)	C4A—N1—C4B—C5B	−7.6 (9)
N1—P2—C31—C32	168.4 (4)	P2—N1—C4B—C5B	−124.3 (10)
C41—P2—C31—C32	55.7 (4)	P1—N1—C4B—C5B	88.3 (12)
Re1—P2—C31—C32	−85.8 (4)	N1—C4B—C5B—C6B	−176.0 (19)
P1—P2—C31—C32	−151.1 (3)	C4B—N1—C4A—C5A	9.0 (10)
N1—P1—C11—C16	−77.7 (4)	P2—N1—C4A—C5A	99.9 (7)
C21—P1—C11—C16	34.9 (5)	P1—N1—C4A—C5A	−103.3 (7)
Re1—P1—C11—C16	174.8 (3)	N1—C4A—C5A—C6A	170.2 (13)
P2—P1—C11—C16	−111.0 (4)		

### Hydrogen-bond geometry (Å, °)

**Table:**

*D*—H···*A*	*D*—H	H···*A*	*D*···*A*	*D*—H···*A*
C12—H12···O3B	0.95	2.51	3.39 (2)	155
C14—H14···Br1^i^	0.95	2.81	3.524 (6)	133
C46—H46···Br1	0.95	2.63	3.519 (6)	157

Symmetry codes: (i) *x*+1, *y*, *z*.
